# Phase II trial of nafamostat mesilate/gemcitabin/S-1 for unresectable pancreatic cancer

**DOI:** 10.1371/journal.pone.0267623

**Published:** 2022-05-11

**Authors:** Tadashi Uwagawa, Taro Sakamoto, Takeshi Gocho, Hiroaki Shiba, Shinji Onda, Jungo Yasuda, Yoshihiro Shirai, Ryoga Hamura, Kenei Furukawa, Katsuhiko Yanaga, Toru Ikegami

**Affiliations:** 1 Department of Surgery, The Jikei University School of Medicine, Tokyo, Japan; 2 Department of Surgery, Fukuoka Sanno Hospital, Fukuoka, Japan; Mayo Clinic in Arizona, UNITED STATES

## Abstract

**Purpose:**

To assess the efficacy of combination chemotherapy with nafamostat mesilate, gemcitabine and S-1 for unresectable pancreatic cancer patients.

**Materials and methods:**

The study was conducted as a single-arm, single center, institutional review board-approved phase II trial. Patients received nafamosntat mesilate (4.8 mg/kg continuous transregional arterial infusion) with gemcitabine (1000 mg/m^2^ transvenous) on days 1 and15, and with oral S-1 [(80 mg/day (BSA<1.25 m^2^), 100 mg/day (1.25 ≤ BSA<1.5 m^2^), or 120 mg/day (BSA ≥1.5 m^2^)] on days 1–14 or, days 1–7 and 15–21. This regimen was repeated at 28-day intervals.

**Results:**

Forty-seven evaluable patients (Male/Female: 31/16, Age (median): 66 (range 35–78) yrs, Stage III/IV 10/37.) were candidates in this study. Two patients in stage III (20%) could undergo conversion surgery. Twenty-four patients (51%) underwent subsequent treatment (1^st^ line/ 2^nd^ line / 4^th^ line, 13/ 10/ 1, FOLFIRINOX: 12, GEM/nab-PTX: 18, TAS-118: 3, chemoradiation with S-1: 2, GEM/Erlotinib: 1, nal-IRI: 1, surgery: 2). Median PFS and OS were 9.7 (95% CI, 8.9–14.7 mo) and 14.2 months (99% CI, 13.3–23.9 mo), respectively. Median PFS in stage IV patients was 9.2 months (95% CI, 8.4–12.0 mo). Median OS in patients without subsequent treatment was 10.8 months (95% CI, 9.1–13.8 mo). Median OS in patients with subsequent treatment was 19.3 months (95% CI, 18.9–31.9 mo). Grade 4 treatment-related hematological toxicities were encountered in 7 patients. Two patients developed grade 3 allergic reaction after 6 cycles or later. No febrile neutropenia has been observed.

**Conclusion:**

NAM/GEM/S-1 therapy is safe and could be promising option for unresectable pancreatic cancer, especially for stage IV cancer.

## Introduction

Pancreatic cancer is the most lethal cancer and the number of the patient is increasing. Currently, pancreatic cancer is the fourth leading cause of cancer-related death in US [1]. Treatment options for advanced pancreatic cancer have increased over the last decade. However, the results of those treatments are not satisfactory. Chemotherapeutic agents-induced nuclear factor-κB (NF-κB) activation plays an important role in chemoresistance [2]. Nafamostat mesilate (NM), a synthetic serine protease inhibitor, inhibits NF-κB activation by inhibiting IκBα phosphorylation and induces apoptosis of pancreatic cancer cells [3]. Furthermore, NM inhibits gemcitabine (GEM) -induced NF-κB activation in pancreatic cancer cells and increases chemosensitivity of GEM [4]. We previously reported clinical studies of GEM in combination with NM for advanced pancreatic cancer patients (median survival; 10 months, 1-year survival rate; 40%) [5, 6]. Recently, we reported phase II study of GEM with NM as an adjuvant chemotherapy for pancreatic cancer [7]. Here, we conducted combination chemotherapy of GEM/NM with S1 Data, oral fluoropyrimidine consisting of tegafur, gimeracil an inhibitor of dihydropyrimidine dehydrogenase and oteracil.

## Material and methods

Chemotherapy-naïve patients with unresectable locally advanced and metastatic pancreatic cancer were eligible for this study. Evaluation of unresectability was assessed using the National Comprehensive Cancer Network guidelines for pancreatic cancer. Other eligibility criteria included the followings: age > 20 and < 80, an Eastern Cooperative Oncology Group (ECOG) performance status score of 0 or 1, adequate bone marrow function (absolute neutrophil count > 1500/mm^3^, hemoglobin > 9.0 g/dl, platelets > 100 000/mm^3^), adequate renal (serum creatinine ≦ 1.0 × upper limit of normal (ULN)) and hepatic function (bilirubin ≦ 2 × ULN, aspartate aminotransferase/alanine aminotransferase ≦ 3 × ULN) was also required. The patients received NM (4.8 mg/kg continuous regional arterial infusion for 24 hrs) with gemcitabine (1,000 mg/m^2^ for 30 min) on days 1 and 15, and oral S-1 [(80 mg/day (BSA<1.25 m^2^), 100 mg/day (1.25BSA<1.5 m^2^) or 120 mg/day (BSA1.5 m^2^)] on days 1–14 or, days 1–7 and 15–21. The treatment was repeated at 28-day intervals. A port-catheter system was implanted in the subcutaneous tissue of the thigh before the treatment. A catheter was inserted in the celiac artery. An appropriate distribution of an iodine contrast agent in the pancreas was confirmed by computed tomography. Patients failed to complete one cycle of this treatment were excluded from the evaluation. Patients with replaced common hepatic artery were also excluded on the ground that NM could not delivered to the liver at an effective concentration. The primary end points in this study were to assess progression-free survival (PFS) and overall survival (OS). As pathological tumor response is not necessarily correlated with imaging tumor response in pancreatic cancer, response rate was not used to evaluate tumor status in this study. The secondary end point was to evaluate safety of the treatment. PFS and OS were estimated with the Kaplan-Meier survival analysis. The incidence and severity of adverse events was evaluated by the Common Terminology Criteria for Adverse Events v5.0. This study was approved by the institutional review board of the Jikei University School of Medicine (Tokyo, Japan). This study was registered at University hospital Medical Information Network (UMIN) clinical trials registry in Japan (UMIN000008413).

### Statistical analysis

The endpoints of this study are PFS and OS, and the Kaplan-Meier method estimates the median survival time and their 95% confidence intervals. The confidence interval of the median survival is statistically calculated on the assumption of asymptotic normality. However, a minimum of 20 cases is required to meet this assumption [8]. Statistical analysis was performed using Statcel 4 on Excel.

## Results

### Patient characteristics

Between August 2011 and December 2018, 47 out of 50 patients were evaluated in this study. Three patients were excluded after enrollment. One patient developed grade 3 rash caused by S-1 in first cycle. One patient requested discontinuation of this treatment during 1 cycle. The catheter could not be placed technically in a patient. The characteristics of the patients are listed in Table 1. About half of the patients underwent subsequent therapy after disease progression. The fact that only GEM and S-1 could be used for pancreatic cancer patients until 2014 in Japan caused the low subsequent treatment rate. In fact, most of the patients who enrolled in 2014 or later underwent subsequent therapy.

**Table 1:** Characteristics of patients (n = 47).

Age (y), median (range)	66, 35–78
Sex	
Male	31
Female	16
TMN staging	
III	10
IV	37
Subsequent therapy, n (%)	
+	24 (51%)
-	23 (49%)
Subsequent therapy, line (%)	
1st	13 (54%)
2nd	10 (42%)
3rd	0 (0%)
4th	1 (4%)
Site of metastasis, n	
Liver	27
Lung	7
Peritoneum	8
Distant lymph nodes	6
The others	4
Treatments, n	
GEM/nab-PTX	19
FOLFIRINOX	12
TAS-118	3
Chemoradiation with S-1	2
GEM/Erlotinib	1
Nal-IRI/FL	1

Nal-IRI: nanoliposomal irinotecan, FL: 5-fluorouracil/ Leucovorin.

### Safety

As for hematologic toxicity, 32 patients (68%) developed grade 3 neutropenia, anemia, thrombocytopenia, serum AST/ALT increased, or creatinine increased. And 7 patients (15%) developed grade 4 (neutropenia: 6, creatinine increased: 1) (Table 2). The patient with Grade 4 creatine increase could receive the subsequent treatment after recovery from renal failure. With regard to no hematologic toxicity, 2 patients developed grade 3 serum sickness after 6 cycles or later. As those patients have recovered from serum sickness after steroid therapy, chemotherapy was continued with the others regimen. Port-catheter system-related adverse event was not observed.

**Table 2:** Treatment-related Adverse events (AEs).

	Grade 3	Grade 4
Event, n (%)		
Hematologic AEs		
Neutropenia	19 (40)	6 (13)
Anemia	3 (6)	0
Thrombocytopenia	2 (4)	0
Serum AST/ALT increased	7 (15)	0
Creatinine increased	0	1 (2)
Hyperkalemia	0	0
Febrile neutropenia	0	0
Nonhematologic AEs		
Fatigue	0	0
Serum sickness	2 (4)	0

### Responses

**Fig 1:**
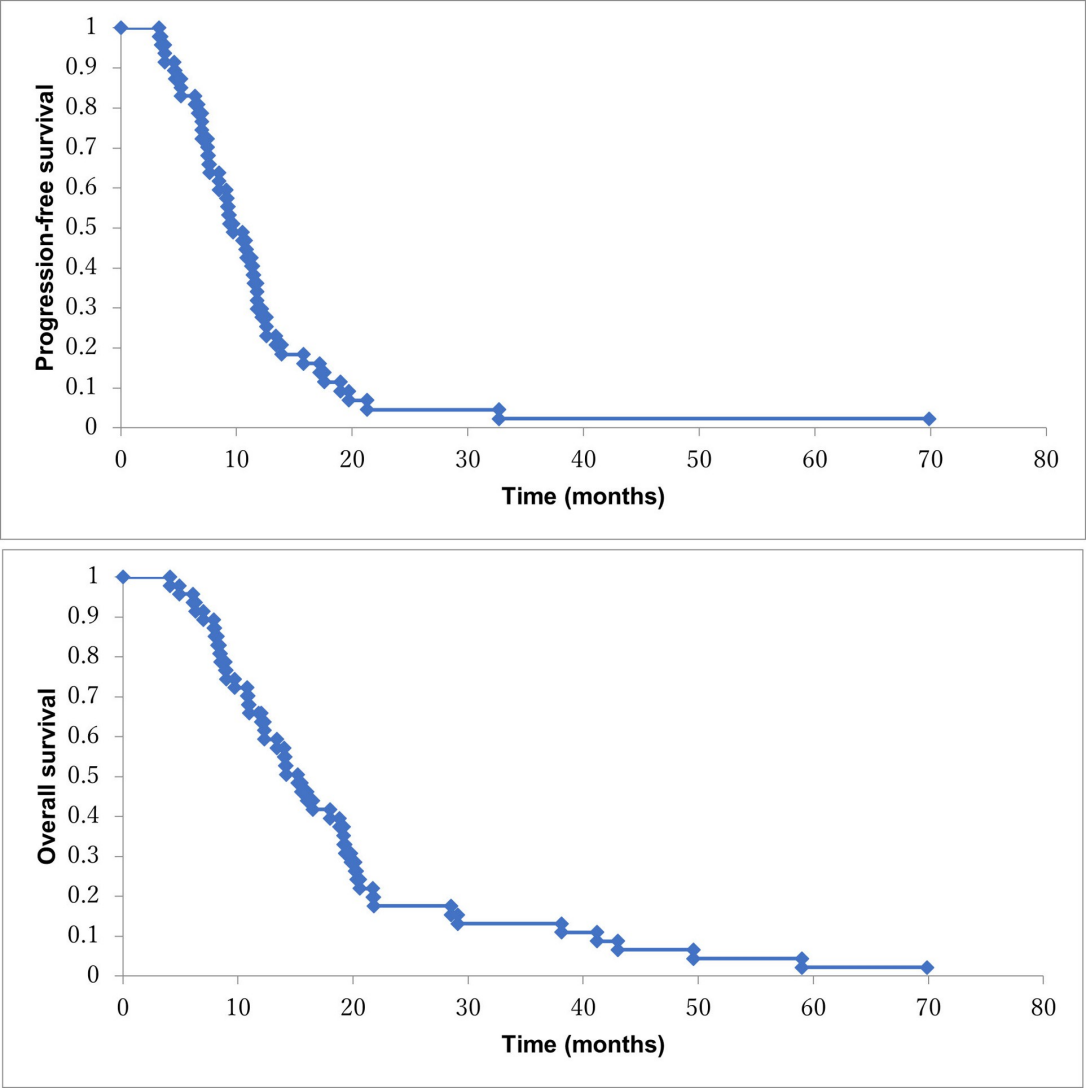
a: Progression-free survival in the all patients treated with NM/GEM/S-1 (n = 47). b: Overall survival in the all patients treated with NM/GEM/S-1 (n = 47).

**Fig 2:**
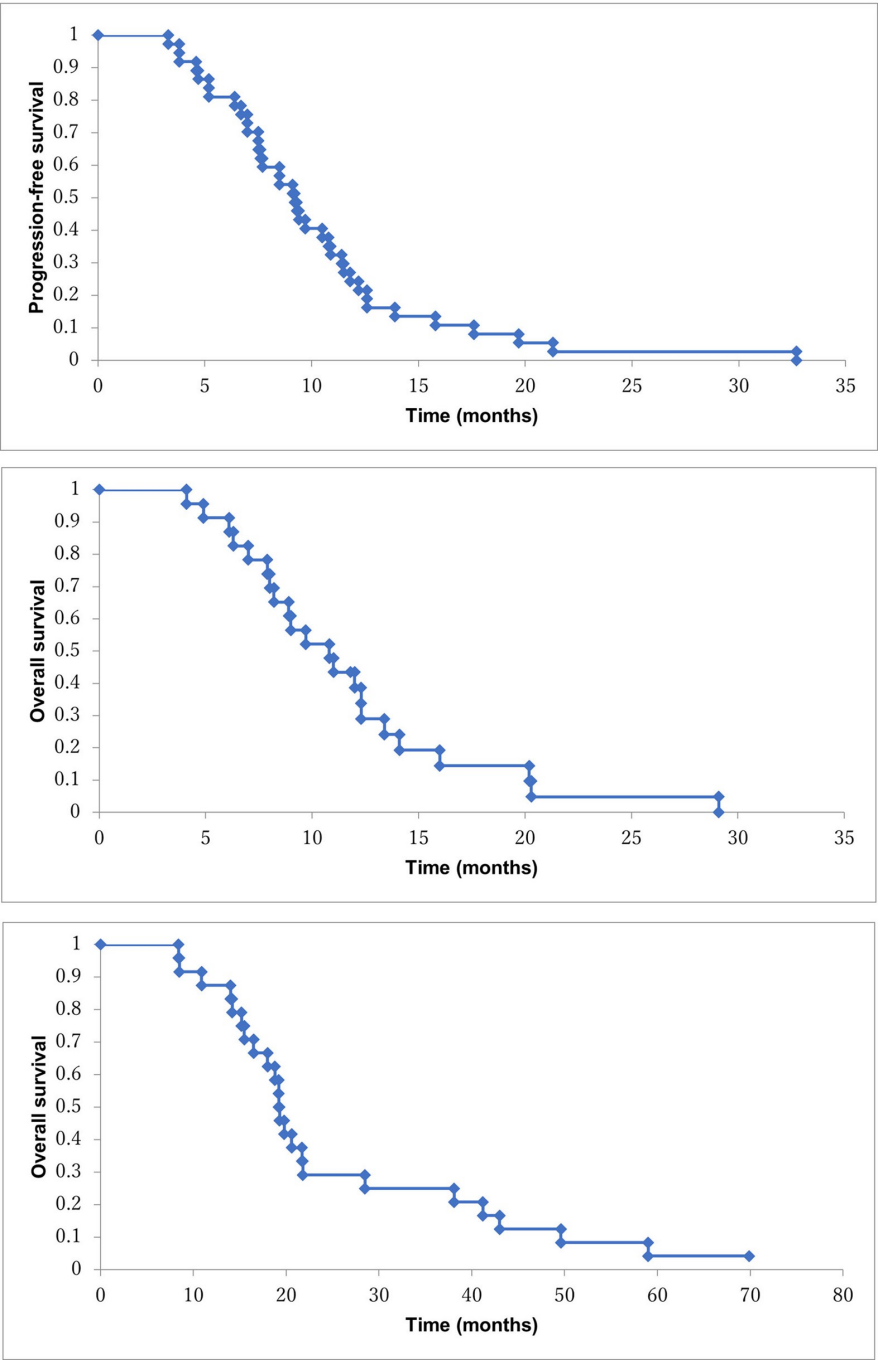
a: Progression-free survival in the stage IV patients with NM/GEM/S-1 (n = 37). b: Overall survival in the stage IV patients with NM/GEM/S-1 (n = 23). c: Overall survival in the patients with sequential treatment (n = 24).

The median PFS and OS were 9.7 (95% CI, 8.9–14.7 mo) and 14.2 months (99% CI, 13.3–23.9 mo), respectively (Fig 1A and 1B). On the other hand, the median PFS and OS in patients without subsequent treatment in stage IV patients were 9.2 months (95% CI, 8.4–12.0 mo) and 10.8 months (95% CI, 9.1–13.8 mo), respectively (Fig 2A and 2B). Median OS in patients with subsequent treatment was 19.3 months (95% CI, 18.9–31.9 mo) (Fig 2C). One-year survival rate in all patients and patients without subsequent treatment were 64% and 39%, respectively (Figs 1A and 2B). Three patients with locally advanced cancer could achieve conversion surgery. Median OS in 3 patients was 43 months (range, 15.5–78.8 mo). The patient with the longest survival is alive without recurrence.

## Discussion

According to National Comprehensive Cancer Network Guidelines (ver 2.2021), chemotherapy with FOLFIRINOX [9] and GEM with albumin-bound paclitaxel [10] (GEM/nab-PTX) were mainly recommended as systemic therapy for advanced pancreatic cancer. Recently, the efficacy of pembrolizumab in patients with noncolorectal high microsatellite instability/mismatch repair-deficient cancers was demonstrated [11]. Olaparib is also an effective agent in certain patients under certain circumstances with germline *BRCA1/2* mutations [12]. However, the indication of those agent is limited. Immune checkpoint blockage is not always effective for tumors with low tumor mutational burden such as pancreatic cancer [13]. New effective treatments have not been reported since the report of GEM/nab-PTX in 2013.

Transcriptional factor NF-κB plays pivotal roles in cancer differentiation and proliferation [14, 15]. Moreover, NF-κB is also involved in chemoresistance [16, 17]. In most pancreatic cancer, NF-κB is activated constitutively [18, 19]. And most cytotoxic agents induce NF-κB activation in pancreatic cancer cells [1]. Therefore, targeting NF-κB could be a reasonable strategy for pancreatic cancer. GEST study did not prove the superiorty of combination therapy of GEM with S-1 compared with GEM alone therapy in OS of inoperable advanced pancreatic cancer [20]. However, inhibition of NF-κB by NM could be expected to improve chemosensitibity of GEM/S-1 therapy. Chemotherapy with NM/GEM/S-1 is safe and leads to an advantage in medical economics [5]. The subsequent treatments after NM/GEM/S-1 definitely resulted in prolongation of survival. Most of patients who were enrolled in this study after 2014 could undergo subsequent therapy, suggesting that NM/GEM/S-1 might not deteriorate performance status. As the regimen of NM/GEM/S-1 does not include effective agents for pancreatic cancer such as platinum-containing drug, irinotecan and taxanes, those agents could be used for the subsequent chemotherapy. As NM, a serine protease inhibitor, is not cytotoxic agent, the adverse event profile is different from that of anticancer drug. NM rarely induces hyperkalemia, no patients developed hyperkalemia in this study. The limitation of this study is small number of patients and the patients with replaced common hepatic artery were excluded. PFS in stage IV patients in this study is acceptable to some extent. Although PFS in all patients is not always acceptable, 3 of 10 patients with locally advanced cancer could achieve conversion surgery, suggesting that NM/GEN/S-1 could contribute to increase the rate of conversion surgery in patients with locally advanced pancreatic cancer.

## Conclusions

Our data showed that an additional treatment based on different therapeutic mechanisms could contribute to prolongation of survival. The treatment with NM/GEM/S-1 could be a novel option for pancreatic cancer, especially for stage IV cancer.

## Supporting information

S1 Data (XLSX)
